# Neoantigen landscape in metastatic nasopharyngeal carcinoma

**DOI:** 10.7150/thno.53229

**Published:** 2021-04-19

**Authors:** Mei Lin, Xiao-Long Zhang, Rui You, Qi Yang, Xiong Zou, Kai Yu, You-Ping Liu, Ru-Hai Zou, Yi-Jun Hua, Pei-Yu Huang, Jin Wang, Qi Zhao, Xiao-Bing Jiang, Jun Tang, Yang-Kui Gu, Tao Yu, Gui-Ping He, Yu-Long Xie, Zhi-Qiang Wang, Ting Liu, Si-Yuan Chen, Zhi-Xiang Zuo, Ming-Yuan Chen

**Affiliations:** 1 Department of Nasopharyngeal Carcinoma, Sun Yat-sen University Cancer Center, 651 Dongfeng East Road, Guangzhou 510060, P. R. China.; 2 Sun Yat-sen University Cancer Center, State Key Laboratory of Oncology in South China, Collaborative Innovation Center for Cancer Medicine.; 3 Guangdong Key Laboratory of Nasopharyngeal Carcinoma Diagnosis and Therapy, Guangzhou 510060, China.; 4 Department of Ultrasound, Sun Yat-sen University Cancer Center, 651 Dongfeng East Road, Guangzhou 510060, P. R. China.; 5 Department of Musculoskeletal Oncology, Sun Yat-sen University Cancer Center, 651 Dongfeng East Road, Guangzhou 510060, P. R. China.; 6 Department of Neurosurgery, Sun Yat-sen University Cancer Center, 651 Dongfeng East Road, Guangzhou 510060, P. R. China.; 7 Department of Breast Oncology, Sun Yat-sen University Cancer Center, 51 Dongfeng East Road, Guangzhou 510060, P. R. China.; 8 Department of Minimally Invasive Interventional Radiology, Sun Yat-sen University Cancer Center, 51 Dongfeng East Road, Guangzhou 510060, P. R. China.

**Keywords:** nasopharyngeal carcinoma, neoantigens, subtype, metastasis, microenvironment

## Abstract

**Background:** Reportedly, nasopharyngeal carcinoma (NPC) patients with MHC I Class aberration are prone to poor survival outcomes, which indicates that the deficiency of tumor neoantigens might represent a mechanism of immune surveillance escape in NPC.

**Methods:** To clearly delineate the landscape of neoantigens in NPC, we performed DNA and RNA sequencing on paired primary tumor, regional lymph node metastasis and distant metastasis samples from 26 patients. Neoantigens were predicted using pVACseq pipeline. Subtype prediction model was built using random forest algorithm.

**Results:** Portraying the landscape of neoantigens in NPC for the first time, we found that the neoantigen load of NPC was above average compared to that of other cancers in The Cancer Genome Atlas program. While the quantity and quality of neoantigens were similar among primary tumor, regional lymph node metastasis and distant metastasis samples, neoantigen depletion was more severe in metastatic sites than in primary tumors. Upon tracking the clonality change of neoantigens, we found that neoantigen reduction occurred during metastasis. Building a subtype prediction model based on reported data, we observed that subtype I lacked T cells and suffered from severe neoantigen depletion, subtype II highly expressed immune checkpoint molecules and suffered from the least neoantigen depletion, and subtype III was heterogenous.

**Conclusions:** These results indicate that neoantigens are conducive to the guidance of clinical treatment, and personalized therapeutic vaccines for NPC deserve deeper basic and clinical investigations to make them feasible in the future.

## Introduction

Nasopharyngeal carcinoma (NPC), which originates from the epithelium of the nasopharynx, is epidemic in southeast Asia, especially in Guangdong, Guangxi and Hong Kong of China 1-3. Approximately 90% patients have loco-regionally advanced (stage II-IVa, AJCC/UICC 8th edition) NPC at first diagnosis 4, and chemoradiotherapy is recommended as a basic treatment for those patients according to the acknowledged National Comprehensive Cancer network guidelines for head and neck cancer 5. However, 20~30% of NPC patients suffer from metastasis after standard chemoradiotherapy 6-8. Once metastasis occurs, the median overall survival (OS) time is as short as 10-20 months 9. For those patients, a novel promising treatment strategy is urgently needed.

Immune checkpoint inhibitors (ICIs) have revolutionized the therapy strategy in cancer by reinvigorating the potential preexisting anti-tumor immune cells 10-13. Although pilot clinical trials show that ICIs do achieve significant survival improvement in some recurrent or metastatic NPC patients, approximately 60%~70% of patients show no durable response 14-16. Apparently, besides upregulation of immune checkpoint molecules, there are other pivotal immune escape mechanisms that exit in NPC.

Mechanisms of immune escape in cancer can be summarized as loss of immunogenicity, orchestrion of immunosuppressive microenvironment and loss of antigenicity 17. Upregulated expression of immune checkpoint molecules like PD-L1 and secretion of suppressive cytokines like IL-10 are typical examples of loss of immunogenicity. Complete tumor ecosystem includes not only mutated tumor cells but also stromal cells like cancer-associated fibroblasts (CAFs) and infiltrated lymphocytes 18. Intense immune pressure imposed by lymphocytes might select tumor cells with high antigenicity and subsequently influence the intratumor heterogeneity. Consequently, complicated and frequent interplays between tumor cells and immune cells or stroma cells might gradually orchestrate an “immune privilege” microenvironment with increased CAFs, regulatory T cells (Tregs) and myeloid-derived suppressor cells (MDSC) and reduced cytotoxic T cells 19. For instance, active cross-talks between malignant and non-malignant cells promote metastasis through a partial epithelial-to-mesenchymal transition program in head and neck cancer 20. Loss of antigenicity could be achieved by loss of tumor antigens and impair antigen presentation. The majority of tumor antigens are neoantigens. Neoantigens are immunogenic peptides derived from specific mutations in tumor or viral open reading frames, and serves as the unique identification of tumor cells 21. And Recognition of tumor specific neoantigens by immune cells requires intact antigen presentation 22, 23. In the classic process of antigen presentation, aberrant proteins are degraded by proteasome, and then the peptide fragments are delivered into the endoplasmic reticulum via transporters associated with antigen processing (TAP) proteins and bind to the MHC class I peptide-loading complex which were subsequently exported to the cell membrane and recognized by T cell receptor (TCR). MHC class I molecules consist of ɑ chain and β_2_-microglobulin are expressed in almost all somatic cells. Thus, antigens presented by cells generating the aberrant proteins itself is called direct presentation, while antigens presented by dendritic cells (DCs) is called cross-presentations which was reported to engender strong and stable immune response^24^. Those tumor cell clones losing the MHC class I expression or neoantigens tend to escape T-cell recognition and then metastasize. Previously, Prof. Kwok-Wai Lo et al. found that NPC patients with MHC class I aberration are prone to poor survival outcomes, which indicates that loss of antigenicity may represent a pivotal mechanism of immune surveillance escape in NPC 25.

With the advantage of paired samples, preliminary studies found that neoantigen depletion occurred during metastasis 26, 27 and ICIs treatment 28. Studies have also proved that neoantigen-based vaccines achieved wonderful tumor control in refractory melanoma 29, 30. Moreover, the combination of ICIs and neoantigen vaccines could further improve treatment efficacy in melanoma in pilot studies 31, 32. Recently, with the advent of next-generation sequencing technology and algorithms predicting neoantigens in silico, identification of ideal neoantigens for vaccine generation has become faster and more convenient 33-35. It has been reported that the quantity and quality of tumor neoantigens are predictive biomarkers of the clinical response of ICIs in variable cancers 36-40. However, studies portraying the neoantigen landscape of NPC are lacking. To determine the antigenicity status of metastatic NPC and its relationship with survival outcomes, we performed whole exome sequencing (WES)/whole genome sequencing (WGS) and RNA sequencing (RNA-seq) on paired primary tumor, regional lymph node metastasis and distant metastasis samples, identified NPC-specific neoantigens using the pVAC-seq pipeline 41, described its characteristics, and finally unveiled the association between neoantigens and clinical outcomes.

## Methods

### Samples and data collection

Following the approval of this study by the ethics committee of Sun Yat-sen University Cancer Center (SYSUCC) (Guangzhou, China), patients at SYSUCC were recruited between June 1, 2012, and May 1, 2016. All the samples were histologically confirmed as nasopharyngeal carcinoma (NPC) (WHO I, II, or III). The quality of tumor samples was examined by tissue sectioning and hematoxylin & eosin (H&E) staining to estimate the tumor content. Only the highest quality samples with ≥ 30% tumor content were selected for subsequent study. Full clinical characteristics of the sequenced patients are provided in Table S3.

### Nucleic acid extraction and WGS/WES/RNA-seq

Frozen tissues and formalin-fixed and paraffin-embedded samples were pulverized using CryoPrep (Covaris, Woburn, MA) and homogenized in lysis buffer from the AllPrep RNA/DNA/Protein Mini Kit (Qiagen, Valencia, CA). DNA, RNA and protein were isolated from each sample using the respective kits (Qiagen, Valencia, CA) following the manufacturer's protocol.

For WGS, a total of 0.8 μg of genomic DNA with high molecular weight (> 20 kb single band) per sample per patient was used for DNA library preparation. A sequencing library was generated using a TruSeq Nano DNA HT Sample Prep Kit (Illumina, USA) following the manufacturer's recommendations, and index codes were added to each sample. Briefly, the genomic DNA sample was fragmented to a size of ~350 bp by a Covaris sonication system. Then, DNA fragments were end-polished, A-tailed, and ligated with the full-length adapter for Illumina sequencing, followed by further PCR amplification. After PCR products were purified (AMPure XP system), libraries were analyzed for size distribution by the Agilent 2100 Bioanalyzer and quantified by real-time PCR (3 nmol/L). Clustering of the index-coded samples was performed on a cBot Cluster Generation System using a HiSeq X PE Cluster Kit v2.5 (Illumina) according to the manufacturer's instructions. After cluster generation, the DNA libraries were sequenced on the Illumina HiSeq X platform, and 150-bp paired-end reads were generated.

For WES, qualified genomic DNA from tumors and matched peripheral blood was fragmented by Covaris technology with resultant library fragments of 180-280 bp, and adapters were then ligated to both ends of the fragments. Extracted DNA was then amplified by ligation-mediated PCR, purified, and hybridized to the Agilent SureSelect Human Exome V6 for enrichment, and nonhybridized fragments were then washed out. Both uncaptured and captured LM-PCR products were subjected to real-time PCR to estimate the magnitude of enrichment. Each captured library was then loaded onto an Illumina HiSeq X platform, and we performed high-throughput sequencing for each captured library independently to ensure that each sample met the desired average fold coverage.

For RNA-seq, 500 ng of total RNA was extracted to prepare RNA libraries using the Illumina TruSeq Stranded Total RNA Kit. Libraries were barcoded and pooled on the Illumina HiSeq X platform.

### SSNV/InDel and SCNA calling from WGS/WES

We used a commercial variant detection pipeline named Sentieon 42 (https://www.sentieon.com), which improves upon BWA-, GATK-, and Mutect- based pipelines, to call somatic single nucleotide variants (SSNVs) and short insertion/deletions (InDels). Based on this pipeline, the 2×150-bp paired-end reads were mapped into the human reference genome (UCSC hg38), and SSNVs and InDels were called after the bam file was sorted and deduplicated.

To further reduce false-positive variant calls, additional filtering was performed. A single-nucleotide variant (SNV) was considered as true positive only if the supported read counts for this SNV ≥ 5, and the p-value calculated by Fisher's test of composition of mutant and wild-type read count between tumor and normal sample should be < 0.05. Variants in variant call format were annotated using ANNOVAR 43.

To detect significantly mutated genes, we first filtered mutations frequently detected (minor allele frequency > 0.001) in normal databases, including the 1000 Genome (2015 Aug, http://www.internationalgenome.org/), ESP6500 (version esp6500siv2, https://esp.gs.washington.edu/drupal/) and ExAC (version ExAC03, http://exac.broadinstitute.org/) databases. Somatic copy number variants (CNV), status of loss of heterogeneity (LOH) and tumor purity estimation (**Figure S1A**) were detected using Control-FREEC v11.1 44.

### Bulk RNA-seq analysis

The 150-bp paired-end reads from RNA-seq were mapped to the human reference genome (UCSC hg38) using STAR v020201 45. RSEM v1.3.0 46 was then used to perform gene expression quantification. DESeq2 v1.20.0 47 was used to perform differential expression analysis. The log_2_TPM-normalized data were used in the correlation analysis.

### Cancer cell fraction (CCF) estimation of variants

The ABSOLUTE v1.0.6 48 algorithm was used to estimate the tumor sample purity, ploidy, and CCF of each SSNV, InDel and CNV. In line with the recommended best practice, all ABSOLUTE solutions were reviewed by 3 researchers, with solutions selected based on majority vote. In this analysis, variants (SSNVs, InDels and CNVs) were classified as either clonal or subclonal based on the confidence interval of the CCF evaluated by ABSOLUTE. Mutations were defined as clonal if the 95% confidence interval overlapped by 1 and as subclonal otherwise.

### HLA typing and neoantigen prediction

We used blood sequencing data in FASTQ format to type human leukocyte antigen (HLA). HLA-HD v1.2.1 49 was selected for class-I HLA genotyping with default parameters.

To identify neoantigens, we used the pVAC-Seq pipeline v4.0.10 41 with the NetMHC, NetMHCpan and PickPocket algorithms to predict 9 and 10 mers neoepitopes. The lowest predicted binding strength of the three predictors was used to define the binding affinity of neoepitopes. Neoepitopes binding stability were evaluated using NetMHCstabpan v1.0 50. Neoepitopes with binding affinity < 500 nM and fragments per kilobase per million (FPKM) > 1 were predicted as neoantigens. Neoantigens with binding strength < 50 nM were defined as strong affinity, < 150 were medium affinity, while others were weak affinity. To quantify the quality of neoantigens, we calculated the Cauchy-Schwarz index of Neoantigens (CSiN) score 38 and the neoantigen fitness model potential 51 as previously described.

### Neoantigen depletion

Copy number loss-related depletion: All nonsynonymous mutations were annotated as either in a region of copy number loss or not. Then, we calculated the odds ratio comparing nonsynonymous mutations that were neoantigens with nonsynonymous mutations that were not predicted to be neoantigens to determine whether neoantigens were more likely to be in regions of copy number loss.

Transcriptional depletion: All nonsynonymous mutations were annotated as expressed in the RNA-seq data or not, using the definitions above. Then, we calculated the odds ratio comparing nonsynonymous mutations that were neoantigens with nonsynonymous mutations that were not predicted to be neoantigens to determine whether neoantigens were less likely to be expressed.

### T cell receptor (TCR) inference from tumor RNA-Seq data

Identification of TCR complementarity-determining region 3 sequences from T cells present in the sequenced tumor sections was performed using MiTCR v1.0.3 52. Briefly, paired-end FASTQ files were concatenated into a single file using seqtk v1.3 (https://github.com/lh3/seqtk) and run through MiTCR with recommended parameters to optimize extraction from RNA-seq datasets.

### Estimation of the immune cell population

Estimation of the immune cell population, such as CD8 T cells, was performed using CIBERSORT 53 tools online with default parameters (https://cibersort.stanford.edu/).

### Gene set variation analysis

Predominantly, pathway analyses were carried out to evaluate activation of Kyoto Encyclopedia of Genes and Genomes (KEGG) pathways. Then, we applied Gene set variation analysis (GSVA) in the GSVA package (version 1.30.0) 54 to assign pathway activity estimates to each sample.

### Enumeration of Epstein-Barr virus (EBV) DNA

EBV read counts extracted from WES/WGS were performed using BioBloom Tools v2.3.2 55, reporting the number of hits and misses per filter set as well as shared and unique reads.

Plasma DNA was extracted using a QIAamp DNA Blood Mini Kit (Qiagen, Dusseldorf Germany). The concentration of EBV DNA in the plasma was measured using real-time quantitative PCR with primers targeting the BamHI-W region of the EBV genome using an ABI Prism 7700 Sequence Detection Analyzer (Applied Biosystems, FosterCity, CA). Fluorogenic PCRs were set up in a reaction volume of 50 µL using the TaqMan PCR Core Reagent Kit (Da-AN Genetic Diagnostic Center, Sun Yat-Sen University). Each reaction contained 10 µL of 5× buffer (50 mM Tris-HCl, 10 mM MgCl_2_, 250 mM KCl and 1 mg/mL gelatin); 10 pmol of each amplification primer and the corresponding fluorescent probes; 200 µM each of deoxyadinosine triphosphate, deoxycytidine triphosphate, deoxyguanine triphosphate and deoxyuridine triphosphate; 5 units of Ampli Taq Gold, and 5 µL of extracted plasma DNA. Amplifications were performed in an Applied Biosystems 7700 Sequence Detector and then analyzed using the Sequence Detection System software (version 1.6.3) developed by Applied Biosystems. Thermal cycling was initiated with a 10 min denaturation step at 95 °C, followed by 10 cycles at 95 °C for 45 s and 55 °C for 1 min and 30 cycles at 95 °C for 30 s and at 55 °C for 45 s. Duplicate samples were analyzed, and the mean quantity of each duplicate was used for further concentration calculations. Multiple negative blanks were included in every analysis.

### Establishment of the subtype prediction model

To extract the signature of subtypes of the Zhang cohort, we first detected the differentially expressed genes (DEGs) between each two subtypes using a Wilcoxon signed-rank test, and those highly expressed in one subtype compared to other two were used as signatures to build the prediction model. We then randomly selected 2/3 (n = 78) of patients in the Zhang cohort as the training dataset, with the remaining patients in the validation dataset, using the mlbench v2.1-1 R package. Using the caret v6.0-84 R package (https://github.com/topepo/caret/), we selected a random forest algorithm to build the subtype prediction model with 100 resampling iterations in the training dataset. We then applied the prediction model to the validation dataset and assessed its accuracy. After validation, we predicted subtypes of patients in our cohort using primary tumor RNA-seq data. For multiregion samples, the mean of probability would be used to classify subtypes. Similar probability of each subtype indicates unclear classification, so samples with prediction probability < 0.4 were filtered to improve the prediction accuracy. The code used for subtype prediction was available in https://github.com/Meimei2/Prediction_NPC.

### Immunogram score (IGS) calculation of the cancer immunity cycle

Enrichment analyses of anti-tumor T cell immunity, T cell priming and activation (activated dendritic cells), trafficking and infiltration of T cells into tumors, the recognition of cancer cells by T cells, inhibitory cells (Tregs and MDSCs), checkpoint molecule expression, and other inhibitory molecules were performed using signatures previously described 56. The GSVA v1.30.0 54 R package was used to calculate the normalized enrichment score (NES) of Gene Set Enrichment Analysis (GSEA), and the NES was then converted into a z-score (Z). For tumor antigenicity, the z-score of the cancer-predicted neoantigen load was calculated. The IGSs of anti-tumor T cell immunity, tumor antigenicity, T cell priming and activation, trafficking and infiltration of T cells into tumors and the recognition of cancer cells by T cells of each patient were defined as 3 + 1.5×Z, while IGSs of absence of inhibitory cells, absence of checkpoint molecule expression and absence of other inhibitory molecules were defined as 3 - 1.5×Z.

### Statistics

R 3.5.0 was used for all statistical analyses. The Kolmogorov-Smirnov normality test was performed to determine if datasets follow a Gaussian distribution in each comparison. If the data were Gaussian, parametric tests were performed (two-tailed unpaired t-tests, one-way ANOVA with Tukey's correction for multiple comparisons, or Pearson correlation). If the data were non-Gaussian, nonparametric tests were applied (Wilcoxon rank test, one-way ANOVA using Kruskal-Wallis with Bonferroni's correction for multiple comparisons, or Spearman correlation). The results were considered statistically significant when P < 0.05, or a lower threshold when indicated by the appropriate test. Survival analysis was performed using the Kaplan-Meier method. A log-rank test was used to evaluate the significance of the difference between different Kaplan-Meier curves. The hazard ratio was determined using a Cox proportional hazards model. The test used and the statistical significance are reported in each figure and table.

## Results

### Characteristics of neoantigens in nasopharyngeal carcinoma

**Figure 1:**
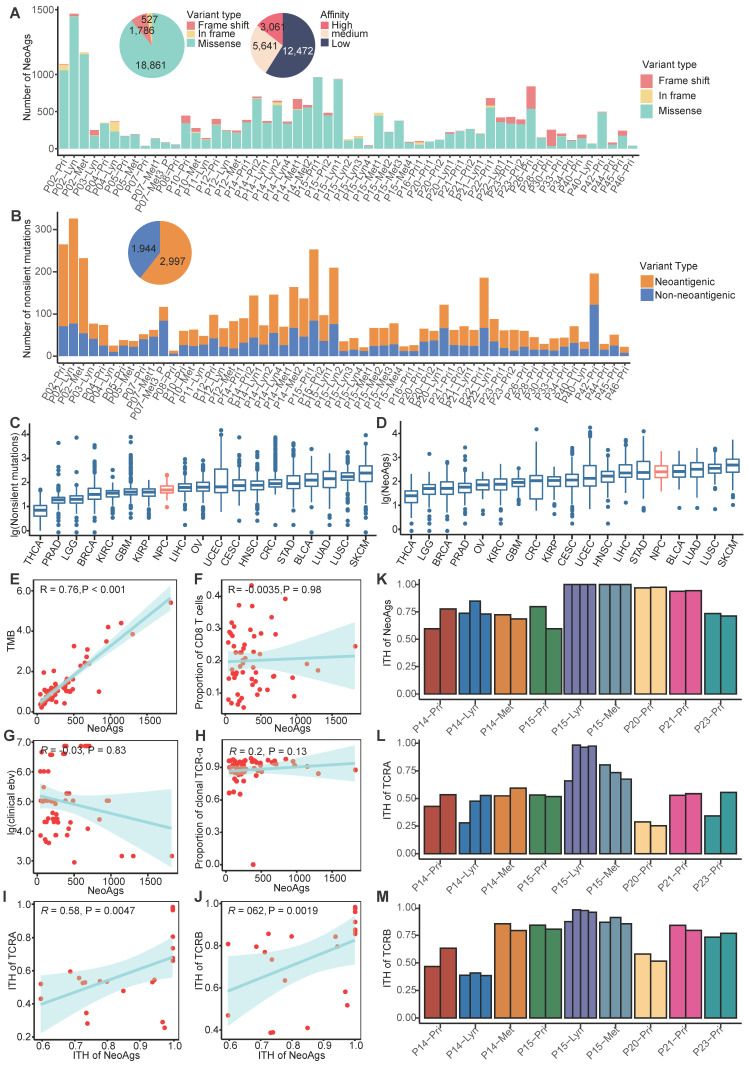
** Landscape of neoantigens in nasopharyngeal carcinoma (NPC). (A)** Bar plot shows the composition of variants types that produced neoantigens. Pie plot on the top left shows the composition of variant types and the different binding affinities of all neoantigens. **(B)** Bar plot and pie plot show the proportions of nonsilent mutations that produced neoantigens. **(C-D)** Comparison of the number of nonsilent mutations (C) and neoantigen load (D) between NPC and other cancers using published The Cancer Genome Atlas program data. THCA: Thyroid carcinoma; PRAD: Prostate adenocarcinoma; LGG: Brain Lower Grade Glioma; BRCA: Breast invasive carcinoma; OV: Ovarian serous cystadenocarcinoma; KIRC: Kidney renal clear cell carcinoma; GBM: Glioblastoma multiforme; UCEC: Uterine Corpus Endometrial Carcinoma; KIRP: Kidney renal papillary cell carcinoma; CRC: Colon adenocarcinoma/Rectum adenocarcinoma Esophageal carcinoma; CESC: Cervical squamous cell carcinoma and endocervical adenocarcinoma; LIHC: Liver hepatocellular carcinoma; HNSC: Head and Neck squamous cell carcinoma; STAD: Stomach adenocarcinoma; NPC: nasopharyngeal carcinoma; LUAD: Lung adenocarcinoma; BLCA: Bladder Urothelial Carcinoma; LUSC: Lung squamous cell carcinoma; SKCM: Skin Cutaneous Melanoma. **(E)** Correlations between tumor mutation burden (TMB) and neoantigen load. **(F)** Correlations between proportion of CD8^+^ T cells and neoantigen load. **(G)** Correlations between clinical detected EBV DNA and neoantigen load. **(H)** Correlations between proportion of clonal TCR-α and neoantigen load. **(I-J)** Correlations between intratumor heterogeneity (ITH) of TCR-α (I) or TCR-β (J) and ITH of neoantigens. **(K-M)** Proportions of branch neoantigens (K), TCR-α (L) and TCR-β (M) of multiregion samples.

To delineate the landscape of neoantigens in NPC, we performed WES/WGS and RNA-seq in 57 samples from 26 NPC patients, including 29 primary tumors, 16 regional lymph nodes and 12 distant metastasis sites (**Table S1-S2**). For available patients (P14, P15, P20, P21, P23), we also included 23 multiregional samples (**Table S1-Table S2**). All samples (except P07-Met3_P) were obtained before treatment. Most patients included in our study were de novo diagnosed metastatic NPC patients; the detailed clinical information is shown in **Table 1**. Following instructions, we detected the 9 and 10 mers MHC class I-associated neoantigens using the pVAC-seq pipeline 41. Neoepitopes with FPKM smaller than 1 or predicting affinity greater than 500 nM were filtered out. In total, we detected 21,174 neoantigens, which included 3,061 high affinity neoantigens (binding affinity < 50 nM) and 1,629 clonal neoantigens (**Figure 1A; Figure S1B; Table S4**; **Supplementary Data**). There were 18,861 neoantigens derived from missense mutations and 1,783 neoantigens from frame-shift insertion/deletions (InDels) (**Figure 1A**). On average, missense mutations generate 4.5 neoantigens per mutation, and frame-shift InDels generate 9.35 neoantigens per mutation, which is comparable to neoantigen data of The Cancer Genome Atlas (TCGA) program 57 (**Table S5**) and is consistent with previous research reported that frame-shift InDels generated more neoantigens than missense mutations did 40. It's worth noted that tumors with relatively low mutations like NPC and thyroid cancer might possess high ability of generating neoantigens (**Table S5**), since the number of predicted neoantigens was also influence by HLA types, binding affinity and expression levels beside number of mutations. In NPC, most neoantigens originated from missense mutations (**Figure 1A**). As expected, although most nonsilent mutations lead to amino acid sequence changes, only approximately 57.33% (23.08%-79.03%) can generate neoantigens, neglecting whether these neoantigens can be recognized by antigen-presenting cells and stimulate a T cell response (**Figure 1B; Figure S2A**).

Compared to the mutation data for tumors in TCGA program, the number of nonsilent mutations of NPC (median = 65) is relatively low and comparable to kidney renal papillary cell carcinoma (KIRP, median = 64) and Liver hepatocellular carcinoma (LIHC, median = 72) (**Figure 1C; Figure S2B-C**). We also compared the neoantigen load of NPC with other cancers using published TCGA data 57. The number of neoantigens of NPC (median = 251) is relatively high and comparable to stomach adenocarcinoma (STAD, median = 235) and lung adenocarcinoma (LUAD, median = 321) (**Figure 1D; Figure S2D-E**). In many other tumors, previous studies found that the neoantigen load was positively correlated with tumor mutation burden (TMB) and infiltration of T cells 58, 59. In NPC, we found that the neoantigen load was positively correlated with TMB (R = 0.76, P < 0.001), but not correlated with proportion of CD8^+^ T cells or CD4^+^ T cells (**Figure 1E-F; Figure S2F**). NPC is closely associated with EBV infection, which has been found to promote genome instability 60, 61. Thus, we hypothesized that more severe EBV infection might be linked to more neoantigens. However, in our study, either the clinically detected EBV DNA copy number or the EBV infection quantified using BioBloom 55 showed no correlation with the neoantigen load (**Figure 1G; Figure S2G**).

Once TCRs recognize antigens, T cells will be activated to destroy the specific enemy. It is rational to hypothesize that tumor neoantigen load is associated with TCR diversity. Therefore, we used MiTCR^52^ to extract and quantify the TCR diversity from RNA-seq data. However, we found that the neoantigen load was not significantly correlated with the number of TCR clones (**Figure 1H; Figure S2H**). Since the extent of TCR selection and clonal expansion is an important indicator of local T cell activation and replication, this phenomenon indicates that due to posttranslational modification or other reasons, many predicted neoantigens cannot stimulate T cells. We further explored the relationship between intratumor heterogeneity (ITH) of neoantigens and ITH of TCR using multiregion samples. We defined the ITH of neoantigens as the proportion of branch neoantigens that are not shared by all samples from different regions 62. Similarly, the proportion of branch TCR represents the ITH of TCR. We observed that ITH of neoantigens was positively correlated with ITH of TCR (**Figure 1I-M**). It indicated that ITH of neoantigens might influence the function of T cells. We further explored genes with high ability of generating neoantigens across all samples. *TP53*, *KIF1A*, *SUN1*, *ARID1B* and *KCNMA1* were found to be able to generate more neoantigens compared to the others (**Figure S2I**). Across all predicted neoantigens, only 8% (1,697/21,174) were found to be shared by samples from the same patient, and no neoantigen was found to be shared by different patients. For example, *TP53* a well-known driver gene reported in NPC 63, was mutated in 31% samples, but it generated distinctly different neoantigens in two samples. It is difficult to identify ubiquitous neoantigens that can serve as biomarkers or treatment targets for NPC (**Figure S2J**).

### Distinct neoantigen characteristics among primary tumor, regional lymph node metastasis and distant metastasis samples

Many studies have confirmed that immune suppression promotes the metastasis of tumor cells 64-67. Since neoantigens are unique identifications of different tumor cell clones, fewer neoantigens are supposed to associate with weaker immune surveillance. Thus, we asked whether neoantigen characteristics were distinct among primary tumors, regional lymph node metastasis and distant metastasis in NPC. Genomic instability is supposed to be correlated with neoantigen load. Firstly, we assessed the weighted genome instability index (wGII) and a threshold of 0.2 accurately distinguished cancer chromosomal instability+(CIN+) from CIN- as previously defined^68^.

**Figure 2:**
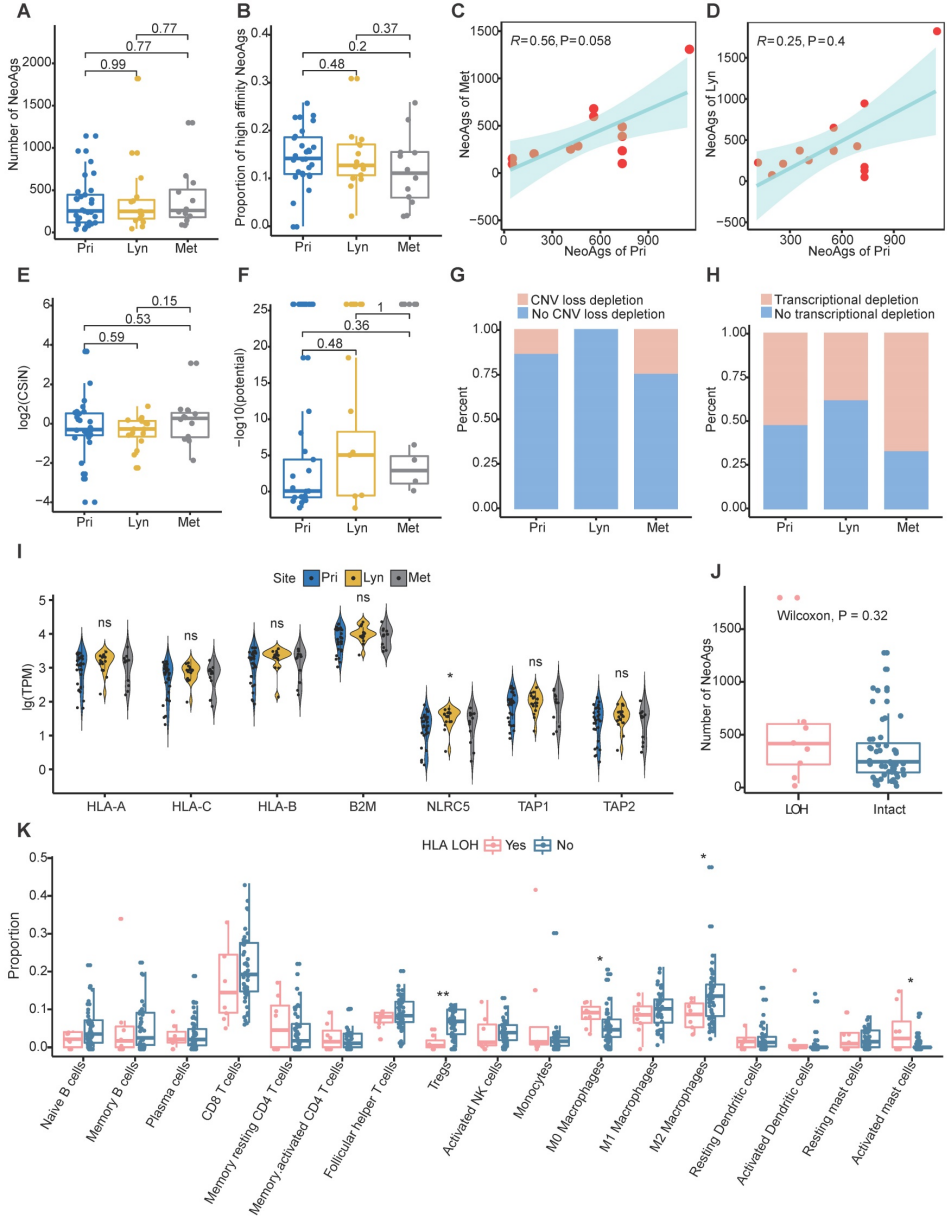
** Characteristics of neoantigens among primary tumors, regional lymph node metastasis and distant metastasis. (A-B)** Neoantigen load (A) and proportion of high-affinity neoantigens (B) were similar among primary tumors, regional lymph node metastasis and distant metastasis. **(C-D)** Scatter plot showing the relationship between the neoantigen loads of primary tumors and distant metastasis (C) or regional lymph node metastasis (D). **(E-F)** CSiN score (E) and fitness model potential of neoantigens (F) were similar among primary tumors, regional lymph node metastasis and distant metastasis. **(G-H)** Bar plot showing the different proportions of copy number loss related (G) and transcriptional (H) neoantigen depletion among primary tumors, regional lymph node metastasis and distant metastasis. **(I)** Violin plot showing the expression of MHC class I related genes among different sites. Wilcoxon signed-rank test: ns: P > 0.05, *: P < 0.05. **(J)** Box plot comparing the number of neoantigens between samples with HLA LOH and those with intact HLA. **(K)** Comparison of proportion of immune cell between samples with HLA LOH and those with intact HLA. Wilcoxon signed-rank test: *: P < 0.05, **: P < 0.01, ***: P < 0.001. Pri: primary tumor; Lyn: regional lymph nodes; Met: distant metastasis.

The CIN status was similar among primary tumor, regional lymph nodes and distant metastasis (**Figure S3A**). And CIN+ tumors tended to possess more neoantigens compared to CIN- tumors although didn't reach the significance probably due to small sample size (**Figure S3B**). In addition, microsatellite instability (MSI) was also assessed using MANTIS 69. All samples were MSI stable, and there was no difference of MSI score among primary, regional lymph nodes and distant metastasis (**Figure S3C**). In addition, the MSI score wasn't correlated with number of neoantigens in NPC (R = 0.099, P = 0.46; **Figure S3D**). Then we compared the neoantigen load and proportion of high-affinity neoantigens among primary tumors, regional lymph node metastasis and distant metastasis. Consist with genomic instability comparison, there were no significant differences in neoantigen load or proportion of high-affinity neoantigens among different sites (**Figure 2A-B**). Additionally, the neoantigen load in primary tumors was positively correlated with the neoantigen load of distant metastasis (R = 0.56, P = 0.058; **Figure 2C**). There was a trend that the neoantigen load at primary sites was correlated with that at regional lymph nodes, albeit without statistical significance (R = 0.25, P = 0.4; **Figure 2D**). Compared with neoantigen load, the neoantigen quality exhibited a more important role in survival outcome prognosis in some cancers 37-39. Therefore, we intended to define the characteristics of the quality of neoantigens among primary tumors, regional lymph node metastasis and distant metastasis. We quantified the quality of neoantigens using the Cauchy-Schwarz index of neoantigens (CSiN) score 38 and neoantigen fitness model potential 51. There were no significant differences in the quality of neoantigens among primary tumors, regional lymph node metastasis and distant metastasis (**Figure 2E-F**). Taken together, our results inferred that the quantity and quality of neoantigens were similar among primary tumors, regional lymph node metastasis and distant metastasis.

Immune suppression promotes tumor metastasis 64-67, and immune suppression has been commonly characterized by neoantigen depletion, which impedes the presentation and recognition of tumor cells. In general, neoantigen depletion may occur at the DNA level through copy-number loss, at the RNA level through suppression of transcripts containing neoantigens, or through posttranslational mechanisms 36. At the DNA level, we quantified the extent of neoantigen depletion due to copy-number loss by comparing the odds ratios between neoantigens and nonneoantigenic, nonsynonymous mutations (**Methods**) 36. And odds ratio greater than 1 means that neoantigens were more likely to occur on genomic segments with copy number loss. At the RNA level, we measured whether neoantigens were preferentially subject to reduction in expression compared to nonneoantigens in the same manner (**Methods**). And odds ratio less than 1 means that transcripts of neoantigens were more likely to be reduced. We found that CNV loss related neoantigen depletion occurred in 13.79% of primary tumors, 0% of regional lymph nodes and 25% of distant metastases, and transcriptional neoantigen depletion occurred in 51.72% of primary tumors, 37.5% regional lymph nodes and 66.67% distant metastases, which indicated that immune suppression characterized by neoantigen depletion might facilitate distant metastasis in NPC (**Figure 2G-H**).

Besides, MHC class I antigen presentation deficiency is also a major tumor escape mechanism from lymphocytes surveillance 23. Previous genomic-wide association study found that single-nucleotide polymorphisms (SNPs) in HLA were independently associated with NPC 70. In the present study, the frequency of mutated MHC class I related genes (including *HLA-A*, *HLA-B*, *HLA-C*, *B2M*, *NLRC5*, *TAP1* and *TAP2*) was relatively low, only two samples (P14-Met2 and P45-Pri) were detected. And the expression of MHC class I related genes except *NLRC5* which was lowest expressed in primary tumor, were similar among primary tumor, regional lymph nodes and distant metastasis (**Figure 2I**). As expected, the expression of *HLA-A* and *HLA-B* were weakly correlated with proportion of clonal TCR-α and TCR-β, respectively (**Figure S3E-F**). Furthermore, we evaluated whether loss of heterogeneity (LOH) happened in HLA genes. LOH of HLA were detected in 8 samples in all, and the frequency of LOH of HLA didn't seem to be correlated with different sites or different type of neoantigens depletion (**Figure S3G**). Samples with HLA LOH seemed to possess higher neoantigens load compare to those with intact HLA, although didn't reach the statistically significance probably due to small sample size (**Figure 2J**). As expected, expression of HLA genes was significantly higher in sample with intact HLA compared to those with HLA LOH (**Figure S3H**). As for immune microenvironment, M0 macrophages and activated mast cells enriched in samples with HLA LOH, while CD8 T cells, Tregs, M1 and M2 macrophages were enriched in samples with intact HLA (**Figure 2K**). Furthermore, we identified four tumor microenvironment immune types (TMITs) based on expression of *CD8A* and *PD-L1* as previous described 71. TMIT I (high PD-L1 and CD8A) represent inflamed tumor that might respond favorably to ICIs, TMIT II (low PD-L1 and CD8A) and TMIT III (high PD-L1 and low CD8A) might represent non-inflamed tumors, while TMIT IV (low PD-L1 and high CD8A) stands for tumors with immune excluded microenvironment 71, 72. In all, 35.1% (20/57), 33.3% (19/57), 15.8% (9/57) and 15.8% (9/57) samples were classified into TMIT I, TMIT II, TMIT II and TMIT IV, respectively (**Figure S3I**). Intriguingly, almost all tumors (87.5%, 7/8) with HLA LOH belongs to TMIT II (**Figure S3I**). All these results indicated that HLA LOH seemed to be related with relatively inactivated immune microenvironment, which might be convenient for tumor cells to proliferate and metastasize.

### Neoantigen reduction happened during metastasis

**Figure 3:**
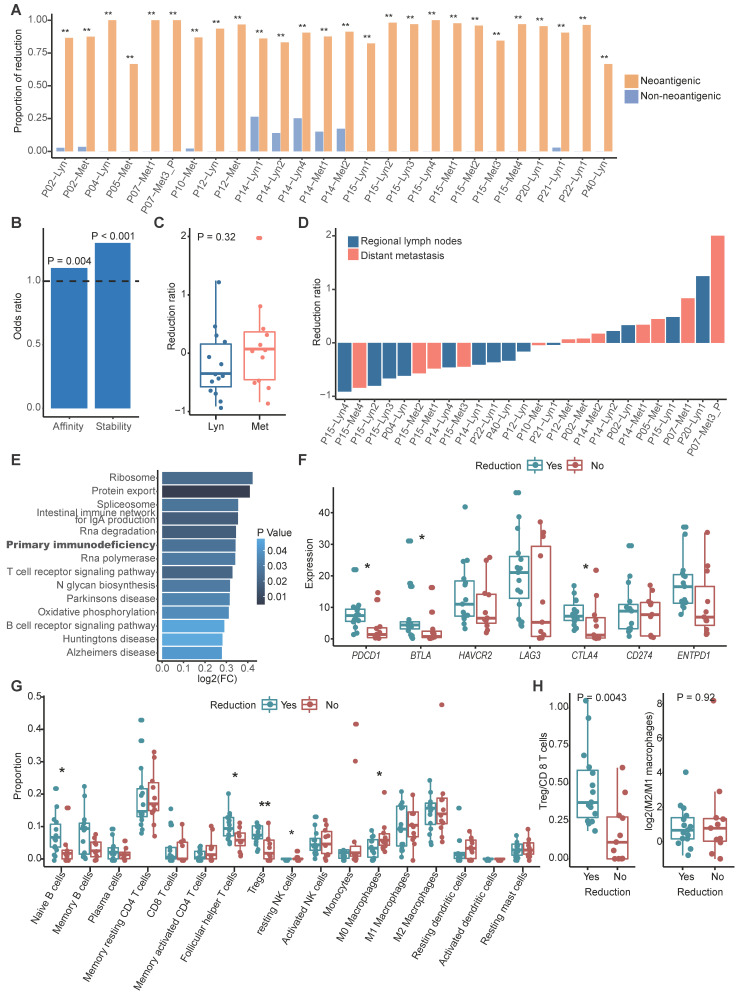
** Neoantigens reduction occurred during metastasis. (A)** Bar plot showing the proportion of the reduced group between neoantigenic mutations and nonneoantigenic mutations. Fisher's exact test: *: P < 0.05, **: P < 0.01. **(B)** Odds ratios of binding affinity and stability compared between the reduced group and increased group. Values > 1 indicates that higher antigenicity neoantigens are more likely to be in the reduced group. **(C)** Distribution of the neoantigen reduction ratio of regional lymph node metastasis and distant metastasis. The Wilcoxon signed-rank test was used. **(D)** Waterfall plot showing the dynamic status of neoantigen reduction of metastatic tumors. **(E)** Results of gene set variation analysis between metastatic tumors with and without neoantigens reduction. **(F)** Box plot shows the expression of immune inhibitor molecules in metastatic tumors with or without neoantigens reduction. **(G)** Comparison of proportion of immune cell between metastatic tumors with and without neoantigens reduction. Wilcoxon signed-rank test: *: P < 0.05, **: P < 0.01. **(H)** Box plot showing the ratio of Tregs/CD8 T cells (left) and M2/M1 macrophages (right) between metastatic tumors with and without neoantigens reduction. The Wilcoxon signed-rank test was used.

With the assumption that tumor cells without clonal neoantigens are privileged to escape immune surveillance and metastasize, we compared the clonality of neoantigens between primary and metastatic sites in paired samples using cancer cell fraction (CCF), which quantifies the clonality of mutations, with CCF = 1 indicating that each tumor cell contains this mutation (**Methods**) 48. According to the dynamic change of CCF, we could categorize neoantigens into three groups: a reduced group, an increased group and a persistent group. The reduced group refers to neoantigens that exist in primary tumors but disappear in metastatic tumors or those that were clonal in primary tumors but subclonal in metastatic tumors. In contrast, the increased group represents neoantigens that were absent in primary tumors but present in metastatic tumors or those that were subclonal in primary tumors but clonal in metastatic tumors. The least common are the persistent group. To determine whether a tumor cell would be prone to discarding mutations that generate neoantigens to escape immune surveillance, we also categorized nonneoantigenic mutations into a reduced group, an increased group and a persistent group, as previously described. As expected, we found that reduction was prone to occur in mutations that produced neoantigens compared to nonneoantigenic mutations during metastasis (**Figure 3A**). It's rational to assume that tumor cells tended to lose neoantigens with high immunogenicity. Binding affinity and stability to MHC class I molecules represent the immunogenicity of antigens, we calculated proportion of strong/medium affinity neoantigens (affinity < 150 nM) and high stability neoantigens (half lift time > 2 h) in the reduced group, as compared to the increased group. Neoantigens with strong immunogenicity are prone to be reduced during process of tumor metastasis (**Figure 3B**).

We then calculated the neoantigen reduction ratio, which was defined as the increased group minus the reduced group and divided by the neoantigen load in primary tumors, to evaluate the extent of neoantigen reduction. We found that neoantigen reduction occurred in 57.69% metastatic tumors (15/26) and tended to occur in metastatic regional lymph nodes (71.43%, 10/14) compared to distant metastasis (41.67%, 5/12) (**Figure 3C-D**). Gene set variation analysis (GSVA) indicated that primary immunodeficiency pathway was enriched in tumors with reduced neoantigens (**Figure 3E**). Immune inhibitors like *PD-1*, *BTLA* and *CTLA-4* were significantly highly expressed in metastatic tumors with reduced neoantigens, which further supported that reduction of neoantigens was associated with functional deficiency of immune cells (**Figure 3F**). Furthermore, B cells and immune regulatory T cells like Tregs and follicular helper T cells were enriched in metastatic tumors with neoantigen reduction, while macrophages and resting dendritic cells were enriched in those without neoantigen reduction (**Figure 3G**). Also, we strived to explore the functional orientation of the immune infiltration via comparing the ratio between Tregs vs CD8 T cells and M2 vs M1 macrophages (**Figure 3H**). As supposed, higher Tregs vs CD8 T cells ratio was observed in metastatic samples with neoantigens reduction compared to those without (**Figure 3H**). Neoantigen reduction which means less antigen stimulation signals might induce the functional suppression of immune cells. The enrichment of immune inhibitors and immune cells indicated that ICIs might obtain supreme tumor control in metastatic tumors with neoantigens reduction.

### Degree of neoantigen depletion is distinct among different subtypes

**Figure 4:**
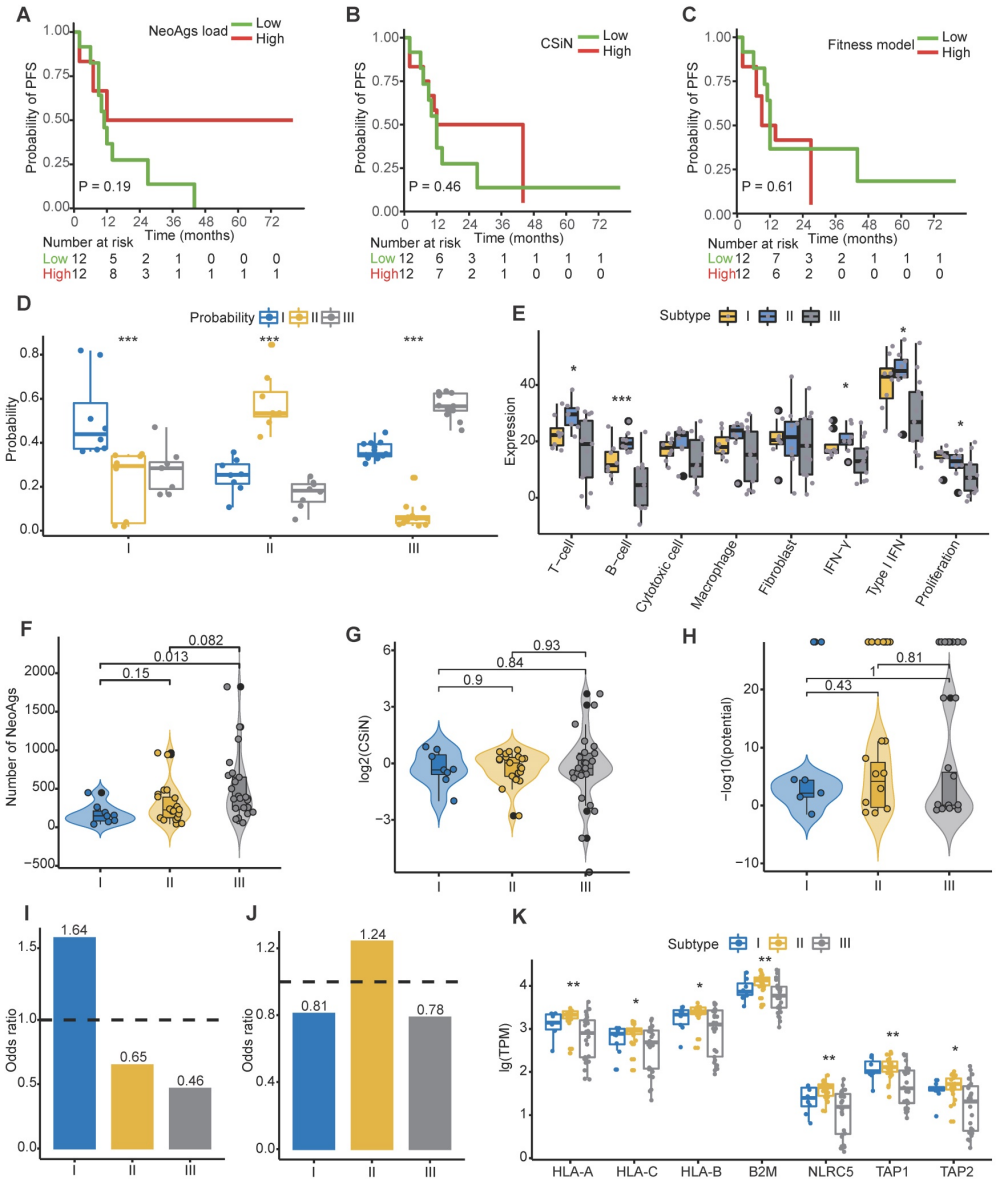
** Signatures of distinct neoantigen depletion among different subtypes. (A)** Kaplan-Meier (KM) curves of progression-free survival (PFS) of patients in high and low neoantigen load groups. **(B)** KM curves of PFS of patients in high and low CSiN score groups. **(C)** KM curves of PFS of patients in high and low neoantigen fitness model potential groups. **(D)** Subtype prediction model probability of different subtypes. ***: P < 0.001, Kruskal-Wallis test. **(E)** Gene signature expression levels (z-normalized) by subtypes. *: P < 0.05, ***: P < 0.001, Kruskal-Wallis test. **(F)** Comparison of the neoantigen loads among different subtypes. **(G-H)** Comparison of the CSiN scores (G) and neoantigen fitness model potential (H) among different subtypes. **(I)** Odds ratios of copy number loss-related neoantigens depletion. Values > 1 indicates that neoantigens are more likely to be in regions of copy number loss compared to nonsynonymous mutations that are not neoantigens. **(J)** Odds ratios of transcriptional neoantigen depletion are shown. Values < 1 indicates that neoantigens are less likely to be expressed compared to nonsynonymous mutations that are not neoantigens. **(K)** Comparison of expression of MHC class I related genes among different subtypes. *: P < 0.05, **: P < 0.01, Kruskal-Wallis test.

Conceivably, tumor cells with abundant neoantigens would be easily recognized and eliminated by the immune system, and studies proved that high TMB or high neoantigen load or high neoantigen quality was associated with favorable treatment response in some cancers 36-38, 40, 73. We found that tumor mutation burden was similar among primary tumor, regional lymph nodes and distant metastasis (**Figure S4A**). When patients were categorized into high and low groups based on the median of TMB of primary tumor, we found that low TMB was associated with worse progression-free survival (PFS) rate (HR = 2.98, 95% CI = 1.1-8.4, P = 0.029), while TMB was not correlated with overall survival (OS) outcome (HR = 3.2, 95% CI = 0.8-12.2, P = 0.074) (**Figure S4B-C**). These results were consistent with previous reported results 74. Next, to determine whether the characteristics of neoantigens can predict survival outcomes of NPC patients, we categorized patients into high and low groups based on the median of neoantigen load, CSiN score and neoantigens fitness model potential, respectively. Unfortunately, we found that neither neoantigen load, CSiN score nor fitness model potential group were associated with patients' PFS or OS (**Figure 4A-C; Figure S4D-F**).

To date, clinicians make therapy strategy decisions mainly based on clinical information, especially tumor stage and the count of EBV DNA segments 5, 75-77. A verified reliable molecular subtype of NPC for application in the clinic is lacking. Using the largest clinical RNA-seq cohort of primary tumors reported to date, Prof. Zhang et al. proposed three molecular subtypes of treatment-naïve NPC mainly based on different immune cell gene expression profiles, but didn't establish the subtype prediction model 78. It has been reported that the PFS of subtype I is worst among the three subtypes, perhaps due to its low immune infiltration and high proliferation. We then asked whether tumor cells from different subtypes also escape immune surveillance through neoantigen depletion. Aiming to answer this question, we built a robust prediction model that could precisely categorize patients into different subtypes. We randomly selected 2/3 patients in the Zhang cohort as the training cohort, and the remaining as the validation cohort. We then built a subtype prediction model based on differential expression genes of the Zhang cohort using a random forest algorithm (**Figure S4G-I; Table S6; Methods**). The prediction accuracy was 85.71%, and the area under receiver operating characteristic (ROC) curve further confirmed the sensitivity and specificity of our model (**Figure S4I**).

We then applied the prediction model to primary tumors of our cohort. For multiregion samples, the mean of the prediction probability would be used to classify subtypes. Samples with a probability less than 0.4 were filtered to improve the prediction accuracy (P14-Pri1, P21-Pri2 and P22-Pri1 were removed). Finally, 17.4% (n = 4), 30.4% (n = 7), and 52.2% (n = 12) patients were categorized into subtype I, subtype II and subtype III, respectively (**Table S3**). The distribution of prediction probability evaluated the accuracy of our prediction model (**Figure 4D**). Consistent with previous reports, patients in subtype II were characterized with high immune infiltration and low proliferation (**Figure 4E**). Using the mutant-allele tumor heterogeneity (MATH) score 79 to quantify ITH, we found that ITH is highest in subtype I and lowest in subtype III among different subtypes (**Figure S4J**). We further explored the neoantigen characteristics among subtypes. The neoantigen load of subtype I was significantly lower than that of subtype III (P = 0.013), while no significant differences in neoantigen quality were detected among subtypes (**Figure 4F-H**). The proportion of high-affinity neoantigens was also similar among subtypes (**Figure S4K-L**). Then we calculated the odds ratio to evaluate the extent of copy number loss related neoantigen depletion and transcriptional depletion in different subtypes. As expected, both CNV loss related neoantigens depletion and transcriptional depletion were severe in subtype I (**Figure 4I-J**). Consistently, expression of MHC class I related genes in subtype II were higher than other subtypes (**Figure 4K**). These results showed that patients in subtype I lacked effective immune cells and suffered from severe neoantigen depletion, which might indicate dismal survival outcomes.

**Figure 5:**
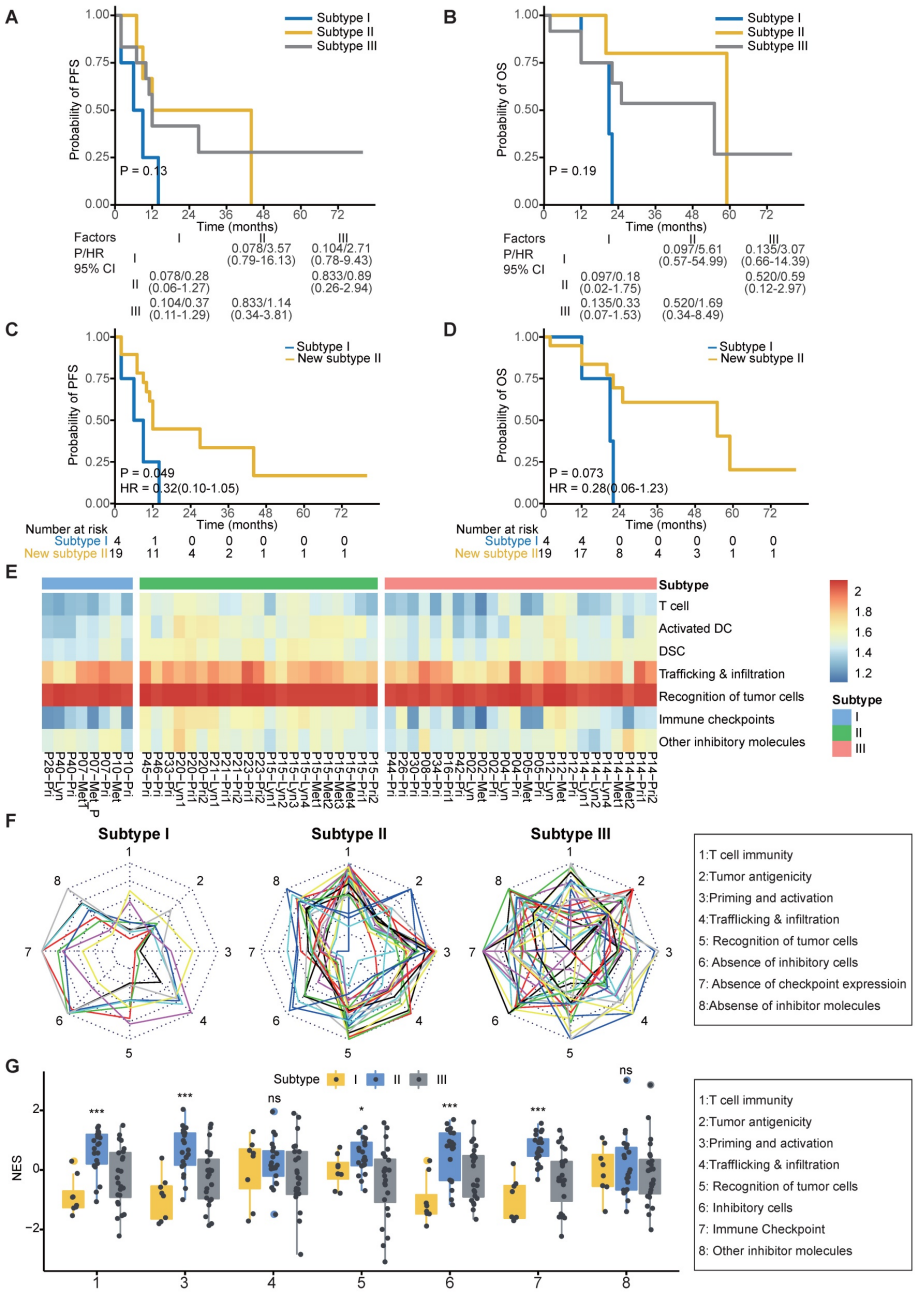
** Clinical outcomes and characteristics of the tumor immunity cycle among different subtypes. (A-B)** Kaplan-Meier curves of progression-free survival (A) and overall survival (B) of patients in different subtypes. **(C-D)** Kaplan-Meier curves of progression-free survival (C) and overall survival (D) of patients in subtype I and new subtype II. **(E)** Heatmap showing the normalized enrichment score of the putative existence of T cell immunity in tumors, priming and activation of T cells, trafficking and infiltration of immune cells, recognition of tumor antigens, enrichment of inhibitor immune cells, expression of immune checkpoint molecules and expression of other immune inhibitor molecules such as IDO1. **(F)** Radar plot of cancer immunity-cycle of tumors from subtype I (left), subtype II (medium) and subtype III (right). Axis 1 represents putative existence of T cell immunity in tumors, axis 2 represents tumor antigenicity (neoantigen load), axis 3 represents priming and activation of T cells, axis 4 represents trafficking and infiltration of immune cells, axis 5 represents recognition of tumor antigens, axis 6 represents absence of inhibitor immune cells such as Tregs, axis 7 represents absence of immune checkpoint molecules such as PD-1 and CTLA-4, and axis 8 represents the absence of other immune inhibitor molecules such as IDO1. The circles from inner to outer represent levels from 1 to 5. **(G)** Box plot comparing the difference of normalized enrichment score (NES) of cancer immunity-cycle of primary tumors among different subtypes.

As expected, patients in subtype I suffered from worse 2-year PFS compared to patients in subtypes II and III (P = 0.049; **Figure 5A-C**). Due to the similar survival between subtypes II and III (**Figure 5A-B**), we merged patients of subtypes II and III as a new subtype II. Patients in subtype I suffered from worse 2-year OS and PFS compared to those in new subtype II (**Figure 5C-D**). Moreover, we found that the merged subtype can better predict PFS (C index: 0.77 vs 0.68) of patients compared to the old one subtype definitions in our cohort.

To help clinicians make precise treatment decisions for each patient, we calculated the immunogram score (IGS) of the cancer immunity cycle, including anti-tumor T cell immunity, tumor antigenicity, T cell priming and activation, trafficking and infiltration of T cells into tumors, the recognition of cancer cells by T cells, absence of inhibitory cells, absence of checkpoint molecule expression, and absence of other inhibitory molecules, as previously reported 56, 80 (**Figure 5E**). We then visualized the cancer immunity cycle status of each sample using a radar plot (**Figure 5F**). We found that patients in subtype I lacked T cells and were weak in tumor antigenicity, antigen presentation and recognition of tumor cells, which might lead to resistance of immunotherapy (**Figure 5G**); thus, novel target treatments for these patients are urgently needed. For example, P40 belonged to subtype I, T cells, tumor antigenicity and recognition of tumor cells were scarce in this patient (**Figure S5A**). Although P40 achieve remission after chemotherapy, tumor progressed and the patient died 21 months later (**Table S3**). In contrast, patients in subtype II highly expressed immune checkpoint molecules (**Figure 5G**), indicating that ICIs might be effective for those patients. For example, P20 from subtype II highly expressed immune inhibitor molecules, and inhibitor cells were enriched in this patient (**Figure S5B**). This patient was free of tumor progression after receiving curative chemotherapy and radiotherapy (**Table S3**). Using ImmuCellAI to predict response of ICIs therapy 81, we found that the proportion of responders was highest in Subtype II (47.6%) and lowest in Subtype I (12.5%) (**Figure S5C**). Subtype III is the most heterogenous group among the three subtypes since no obviously ubiquitous drawbacks were observed directly. Thus, treatment decisions for patients in subtype III need more attention, with comprehensive considerations of clinical information, immune status and molecular subtypes.

## Discussion

Based on DNA and RNA sequencing of paired primary tumor, regional lymph node metastasis and distant metastasis samples, for the first time, we portrayed the landscape of neoantigens in NPC. We found that the neoantigen load of NPC is above average compared to other cancers in TCGA. The quantity and quality of neoantigens is similar among primary tumors, regional lymph node metastasis and distant metastasis, but neoantigen depletion was more severe at metastatic sites compared to primary tumors. Tracking the clonality change of neoantigens during metastasis, we found that neoantigen reduction occurred in metastatic tumors, especially in regional lymph node metastasis. And enrichment of immune checkpoint molecules and immune cells were seen in metastatic tumors with neoantigens reduction. To stratify patients into distinct risk subgroups, we built a subtype prediction model based on previous reported data. Visualizing the tumor immunity cycle using a radar plot, we found that subtype I lacked T cells and suffered from severe neoantigen depletion, subtype II highly expressed immune checkpoint molecules and suffered from the least neoantigen depletion, while subtype III was heterogenous.

The relationship between neoantigen load and survival is controversial. Higher neoantigen load has been associated with better survival in breast cancer 58 and melanoma 82, but worse survival in multiple myeloma 83. For NPC, the neoantigen load fails to predict the clinical survival outcomes of patients. Higher TMB, which is linked to higher neoantigen load, has been associated with response of ICI therapy 84, 85, and tumor antigenicity is a crucial part of the tumor immunity cycle, prompting the hypothesis that a higher neoantigen load is also associated with the efficacy of immunotherapy. Previous studies demonstrated that neoantigen load can predict the response of ICIs 36, 40. However, although the neoantigen load of NPC is comparable to that of LUAD, the response rate of ICIs is lower in that in NPC patients 14-16. Identification of ICI-sensitive patients and the combination of traditional radiotherapy or other target therapies, such as anti-vascular endothelial growth factor receptor treatment, are promising strategies to improve the therapy efficacy of ICIs in NPC, and related preliminary clinical trials are underway (NCT04073784). Building a prediction model with previously published data, we found that patients in subtype II highly expressed immune checkpoint molecules, indicating that ICIs might be effective for those patients, especially for patients with high neoantigen load. Since patients in subtype I lack immune cells and suffer from severe neoantigen depletion, immune therapies are prone to fail in these patients; thus, novel target therapies are urgently needed. In addition, a radar plot of IGS allows clinicians to clearly visualize immunity cycle status and help them make precise treatment decisions.

In addition to ICIs, another important immunotherapy for NPC is therapeutic vaccines. Since the tumorigenesis of NPC is closely associated with EBV, the viral antigens are considered surrogate neoantigens in NPC 1, 86. Low frequency of mutations, an immature neoantigen prediction system, and the high associated expense hinder the development of individualized mutation-based vaccines. Therefore, therapeutic vaccines for NPC have mainly focused on EBV proteins in the past few years, such as autologous DCs incubated with LMP2 peptides 87, total RNA derived from a C666-1 cell line retaining EBV 88, and a fusion protein containing the carboxyl terminus of EBNA-1 fused to LMP2 89. Due to the low immunogenicity of viral antigens and the defective antigen presentation, the efficacy of an EBV vaccine has been limited. With the advent of next-generation sequencing and the development of multiple algorithm to predict the structure and binding affinity of neoepitopes with improved accuracy, it has become possible to customize neoantigen vaccines more quickly and less expensively, which has shown promising therapeutic value in melanoma 29, 32. There are sufficient unique candidate neoantigens for vaccine generation in NPC patients, and personalized vaccines might shed light on patients with sufficient immune cells but low tumor antigenicity. Since the effects of posttranslational modification might influence the biological function of neoantigens, mass spectrometry-based proteomics which was not available in the present study because of the limit of tissue is an important method to verify the stable existence of neoantigens and should be applied in the following researches. Additionally, to design reliable customized vaccines for patients in the clinic, verify whether the predicted neoantigens could stimulate T cells using autologous immune cells is necessary.

Inefficient tumor antigenicity has been proved to be one of the mechanisms of limited ICI efficacy in non-small cell lung cancer 90. Since we observed neoantigen depletion in our study, it is rational to hypothesize that deficiency of tumor antigens might be one of the key mechanisms of ICI resistance in NPC. However, since no patients in our cohort have received ICIs, we failed to define the direct relationship between neoantigen characteristics and the response of ICIs; we will explore this question in our subsequent studies.

In summary, for the first time, we delineated the characteristics of neoantigens in NPC by leveraging multiomics sequencing of paired samples. Compared to primary tumors, metastatic sites suffered from more severe neoantigen depletion. In addition, neoantigen reduction occurred during metastasis. Using a radar plot, we visualized the characteristics of the tumor immunity cycle for each patient to guide clinical decision making. All these results indicate that neoantigens are conducive to the guidance of clinical treatment, and personalized therapeutic vaccines for NPC deserve deeper investigations in basic and clinical studies to make them feasible in the future.

## Supplementary Material

Supplementary figures.

Supplementary table S1.

Supplementary table S2.

Supplementary table S3.

Supplementary table S4.

Supplementary table S5.

Supplementary table S6.

## Figures and Tables

**Table 1:** Clinical characteristics of patients (n = 26)

Characteristics	Entire cohort
Age, Median (IQR)	45.0 (39.0-53.0)
**Gender**	
Male, no. (%)	23 (88.46%)
Female, no. (%)	3 (11.54%)
**Smoking**	
Yes, no. (%)	10 (38.46%)
No, no. (%)	16 (61.54%)
**Pathology (WHO)**	
I/II, no. (%)	0 (0%)
III, no. (%)	26 (100%)
**T stage**	
T1-2, no. (%)	5 (19.23%)
T3-4, no. (%)	21 (80.77%)
**N stage**	
N0-1, no. (%)	3 (11.54%)
N2-3, no. (%)	23 (88.46%)
**M stage**	
M0, no. (%)	6 (23.08%)
M1, no. (%)	20 (76.92%)
**Local-regional radiotherapy**	
Yes, no. (%)	12 (46.15%)
No, no. (%)	14 (53.85%)
**EBV DNA, Median (IQR)**	26,050 (8,438-104,500)
