# Supplementary material for: Neoantigen landscape in metastatic nasopharyngeal carcinoma
Source: Theranostics. 2021 Apr 19;11(13):6427–44. doi: 10.7150/thno.53229 (PMC8120206; doi:10.7150/thno.53229)
Supplement: Supplementary file 1 — Supplementary figures. [file thnov11p6427s1.pdf]

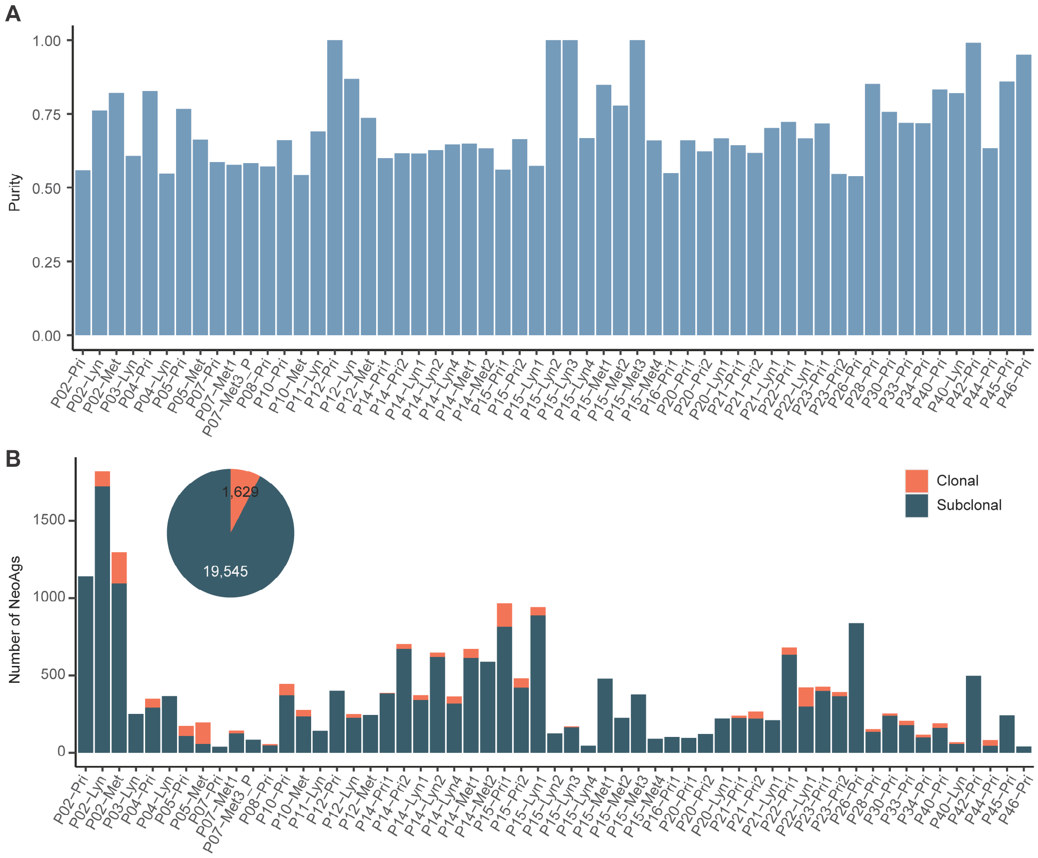

Figure S1, related to Figure 1. Tumor purity and clonality of neoantigens.

(A) Tumor purity estimated using copy number variants.

(B) Bar plot shows the composition of clonal and subclonal neoantigens. Pie plot on the top left shows the composition of clonality of all neoantigens.

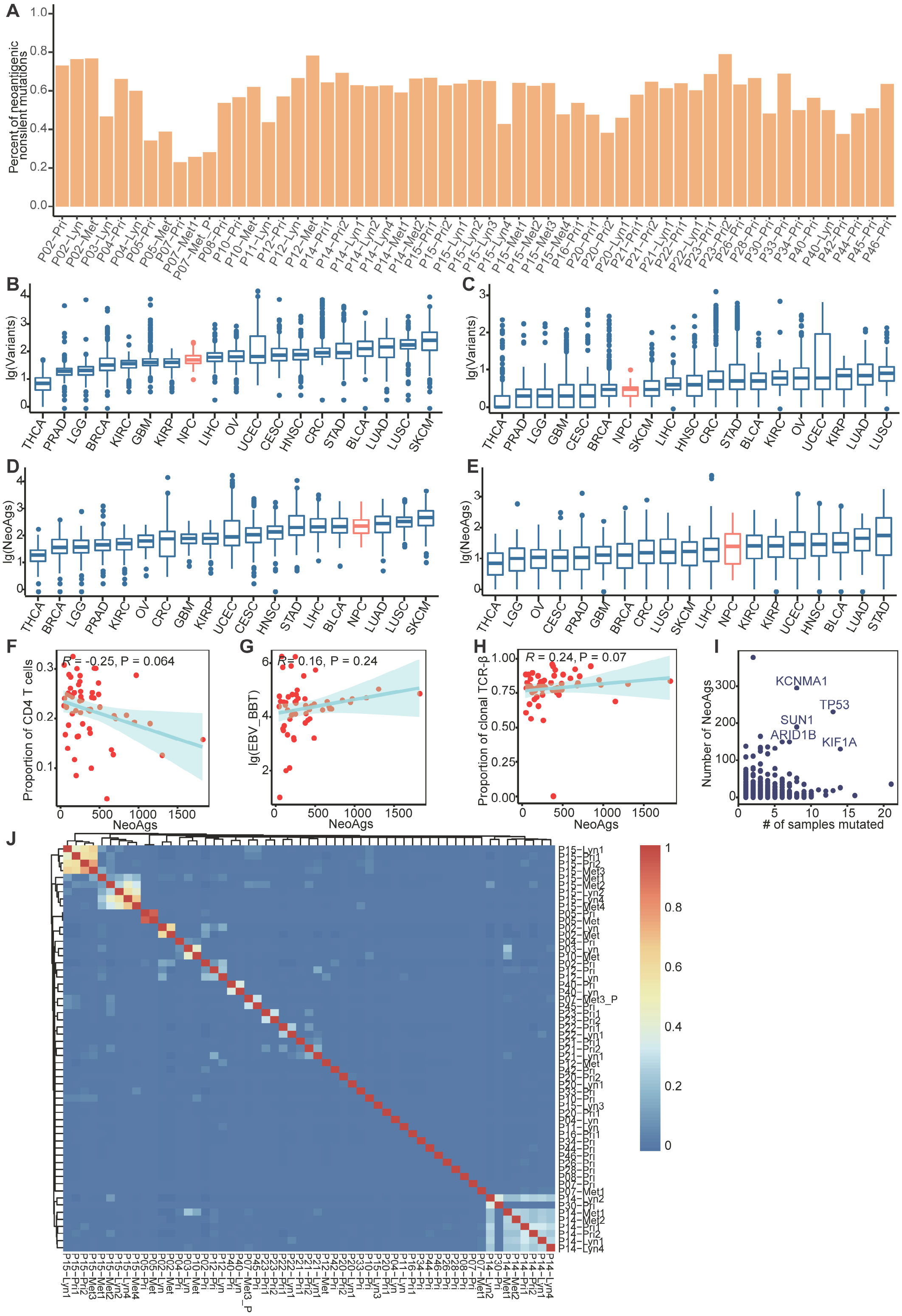

Figure S2, related to Figure 1. Characteristics of neoantigens in nasopharyngeal carcinoma (NPC)

(A) Bar plot shows the proportions of neoantigenic nonsilent mutations.

(B-C) Comparisons of the numbers of missense mutations (B) and frame shift

insertion/deletions (C) between NPC and other cancers using published The Cancer Genome Atlas (TCGA) data.

(D-E) Comparison of the numbers of neoantigens derived from missense mutations (D) and frame shift insertion/deletions (E) between NPC and other cancers using published TCGA data. THCA: Thyroid carcinoma; PRAD: Prostate adenocarcinoma; LGG: Brain Lower Grade Glioma; BRCA: Breast invasive carcinoma; OV: Ovarian serous cystadenocarcinoma; KIRC: Kidney renal clear cell carcinoma; GBM: Glioblastoma multiforme; UCEC: Uterine Corpus Endometrial Carcinoma; KIRP: Kidney renal papillary cell carcinoma; CRC: Colon adenocarcinoma/Rectum adenocarcinoma Esophageal carcinoma; CESC: Cervical squamous cell carcinoma and endocervical adenocarcinoma; LIHC: Liver hepatocellular carcinoma; HNSC: Head and Neck squamous cell carcinoma; STAD: Stomach adenocarcinoma; NPC: nasopharyngeal carcinoma; LUAD: Lung adenocarcinoma; BLCA: Bladder Urothelial Carcinoma; LUSC: Lung squamous cell carcinoma; SKCM: Skin Cutaneous Melanoma.

(F) Correlations between proportion of $CD4^+$ T cells and neoantigen load.

(G) Correlations between BioBloom-predicted EBV infection and neoantigen load.

(H) Correlations between proportion of clonal TCR-β and neoantigen load.

(I) Recurrent genes with high ability of generating neoantigens.

(J) Correlation heatmap based on the number of neoantigens derived from different genes between different samples.

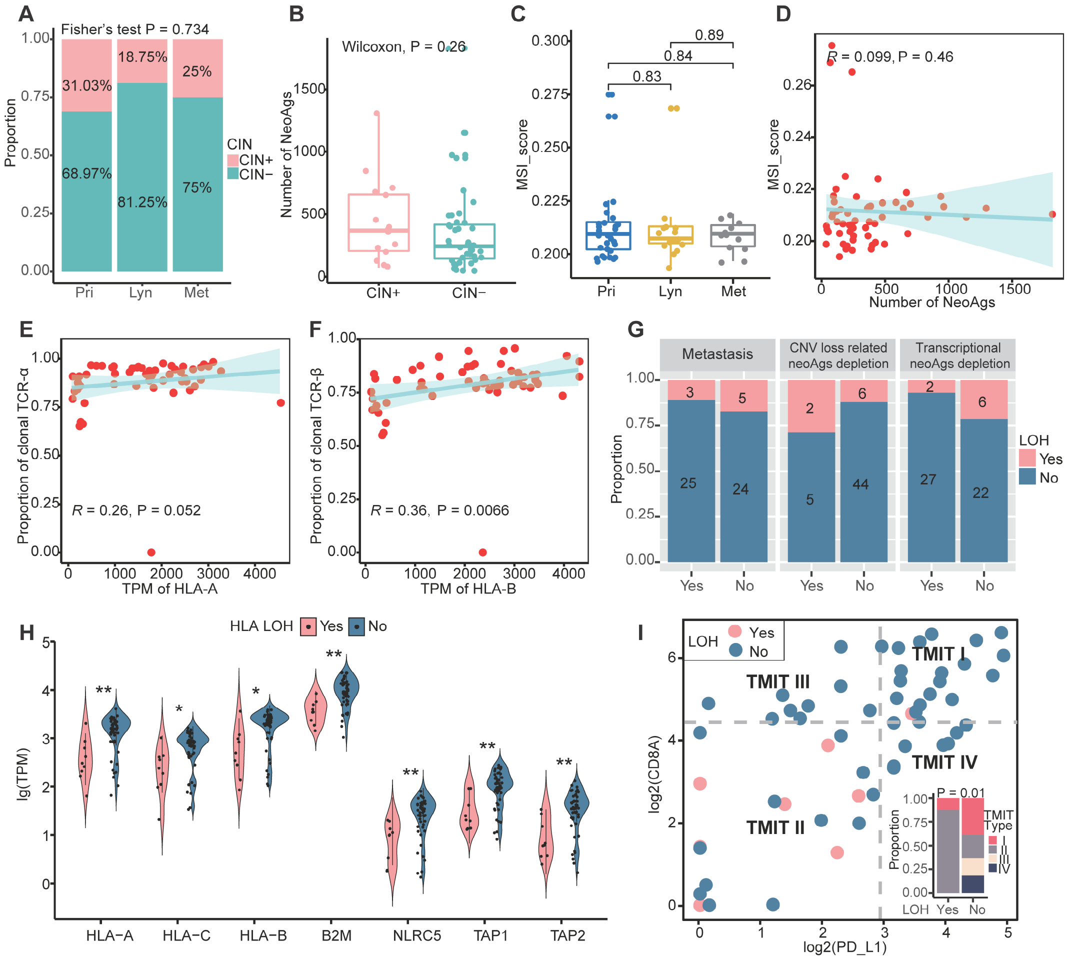

Figure S3, related to Figure 2. Genomic instability and HLA LOH comparison.

(A) Bat plot showing the distribution of cancer chromosomal instability (CIN) status among different sites.

(B) Box plot shows the number of neoantigens between CIN+ and CIN- tumors.

(C) Box plot showing the MSI score of primary, regional lymph nodes and distant metastasis tumors.

(D) Correlations between number of neoantigens and MSI score.

(E) Correlations between Transcripts Per Million (TPM) of *HLA-A* and proportion of clonal TCR-α.

(F) Correlations between TPM of *HLA-B* and proportion of clonal TCR-β.

(G) Bar plot shows the different proportions of metastasis (left), CNV loss related neoantigen depletion (middle) and transcriptional neoantigen depletion (right) between samples with HLA LOH and those with intact HLA.

(H) Violin plot compared the expression of MHC class I related genes between samples with HLA LOH and those with intact HLA. *: $P < 0.05$, **: $P < 0.01$, Wilcoxon signed-rank test.

(I) Scatter plot showing the classification of tumor immune microenvironment types (TIMTs) with bar plot on the left showing the composition of TIMTs of tumors with HLA LOH and those without, each dot represented a sample.

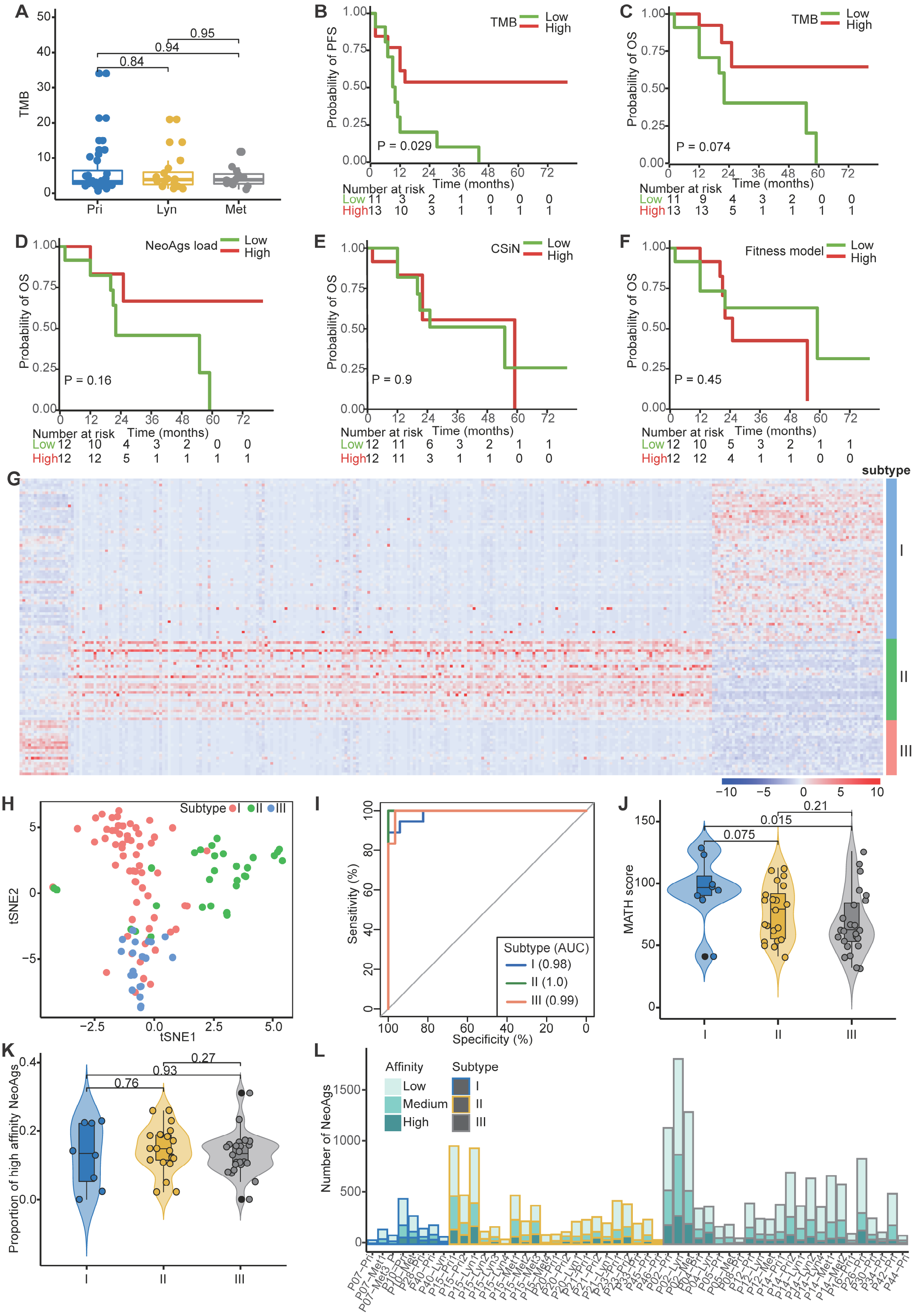

Figure S4, related to Figure 4. Expression and neoantigen characteristics among subtypes

(A) Box plot shows the difference of tumor mutation burden (TMB) among different sites.

(B-C) Kaplan-Meier (KM) curves of progression-free survival (PFS) and overall survival (OS) of patients in high and low TMB groups.

(D) KM curves of OS of patients in high and low neoantigen load groups.

(E) KM curves of OS of patients in high and low CSiN score groups.

(F) KM curves of OS of patients in high and low neoantigen fitness model potential groups.

(G) Heatmap showing the differentially expressed genes (DEGs) among different subtypes in the Zhang cohort. Detailed information can be found in Table S6.

(H) tSNE plot showing the distribution of different subtypes in Zhang cohort based on DEGs.

(I) Receiver operating characteristic curve proving the accuracy of the prediction model.

(J) Comparison of the mutant-allele tumor heterogeneity (MATH) score among different subtypes.

(K) Comparison of the proportion of high-affinity neoantigens among different subtypes.

(L) Bar plot showing the composition of different affinity neoantigens across samples.

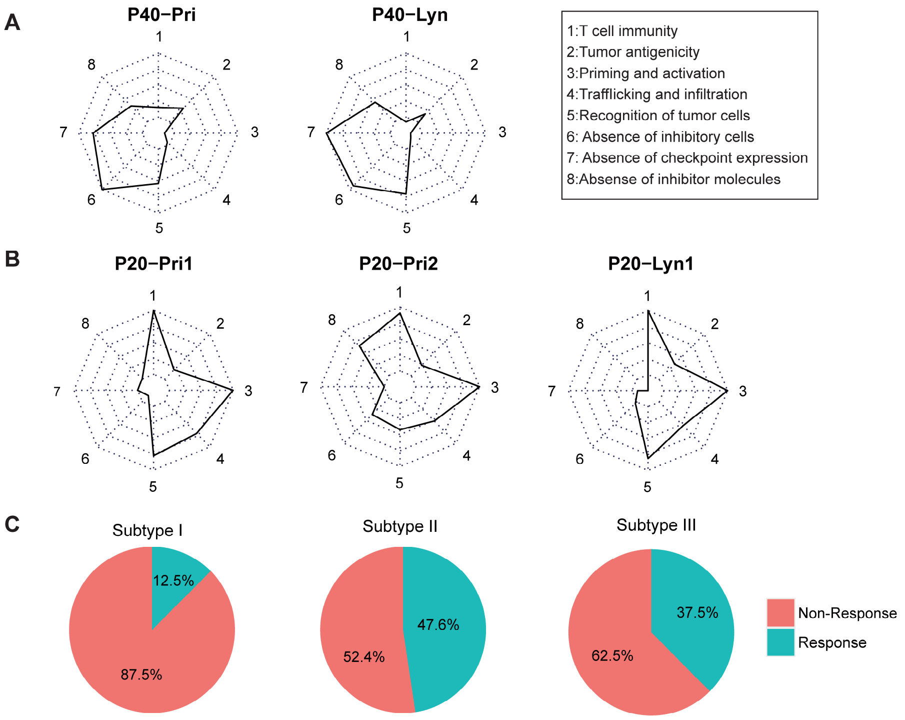

Figure S5, related to Figure 5. Radar plot of the cancer immunity cycle

(A-B) Radar plot of the cancer immunity cycle of P40 from subtype I (A) and P20 from subtype II (B). Axis 1 represents putative existence of T cell immunity in tumors, axis 2 represents tumor antigenicity (neoantigen load), axis 3 represents priming and activation of T cells, axis 4 represents trafficking and infiltration of immune cells, axis 5 represents recognition of tumor antigens, axis 6 represents absence of inhibitor immune cells such as Tregs, axis 7 represents absence of immune checkpoint molecules such as PD-1 and CTLA-4, and axis 8 represents the absence of other immune inhibitor molecules such as IDO1. The circles from inner to outer represent levels from 1 to 5. (C) Pie plot showing the proportion of predicted responders of ICIs therapy among different subtypes.

Table S1. DNA sequencing information of samples.

Table S2. RNA sequencing information of samples.

Table S3. Detailed clinical and subtype information of patients (n = 26).

Table S4. Characteristics of neoantigens in each sample.

Table S5. Average number of neoantigens derived from per missense or Indel mutations in TCGA.

Table S6. Differentially expressed genes of the Zhang cohort.

Supplementary data. Predicted neoantigens of each sample.
